# Segmental Testicular Infarction, an Underdiagnosed Entity: Case Report with Histopathologic Correlation and Review of the Diagnostic Features

**DOI:** 10.1155/2016/8741632

**Published:** 2016-02-14

**Authors:** Sahar Shiraj, Nisha Ramani, Andrij R. Wojtowycz

**Affiliations:** ^1^Department of Radiology, SUNY Upstate Medical University, Syracuse, NY, USA; ^2^Department of Pathology, SUNY Upstate Medical University, Syracuse, NY, USA

## Abstract

A 30-year-old male presented with a 1-day history of left scrotal pain and a tender left testicle and epididymis on physical exam. Scrotal ultrasound showed an avascular, heterogeneous, hypoechoic lesion in the superior left testis suggestive of infarction or neoplasm. The patient was managed conservatively; however, his pain continued and follow-up ultrasound 6 days later showed interval increase in the size of the mass. Left radical orchiectomy was done and pathology result showed segmental infarction of the left testis.

## 1. Introduction

Segmental testicular infarction is a rare condition that presents with acute scrotal pain and is often clinically indistinguishable from other etiologies of scrotal pain. The diagnosis often relies on imaging studies, with testicular neoplasm being the most important differential diagnosis. If the condition is diagnosed or suspected on imaging studies, patients can be managed conservatively and often improve. However, orchiectomy to obtain pathologic diagnosis may be required in a significant number of patients when the diagnosis is uncertain. We present a patient with segmental scrotal infarction who did not improve with conservative management and subsequently underwent orchiectomy.

## 2. Case Presentation

**Figure 1:**
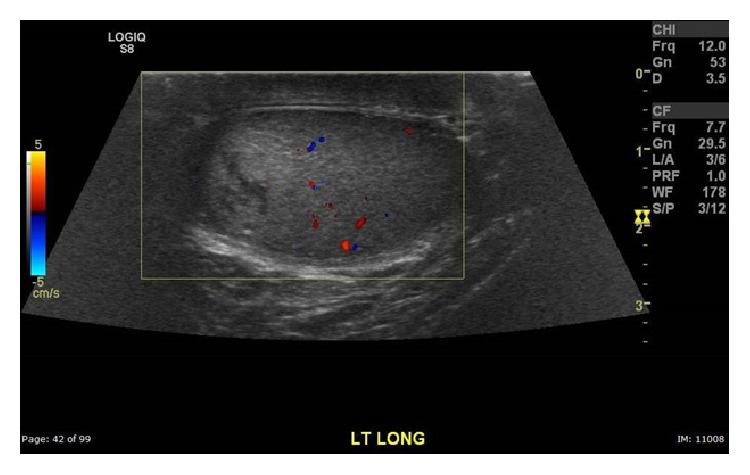
Color-Doppler ultrasound image of the left testis shows an avascular echogenic lesion with hypoechoic center.

**Figure 2:**
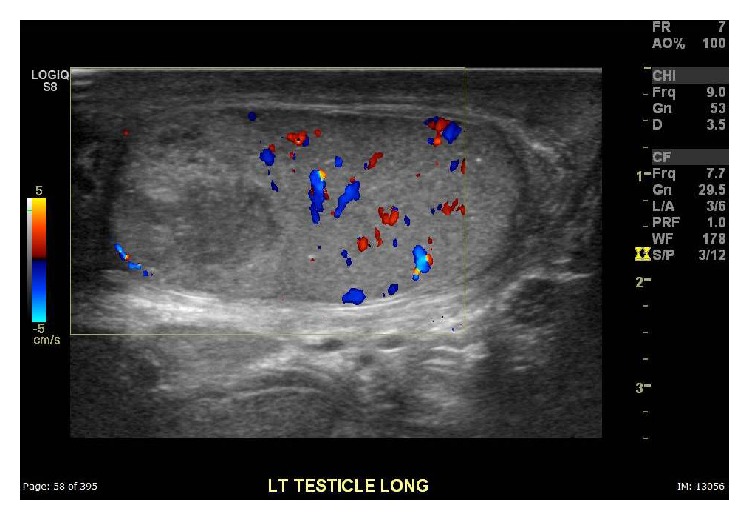
Follow-up color-Doppler ultrasound image shows interval enlargement of the testicular lesion.

**Figure 3:**
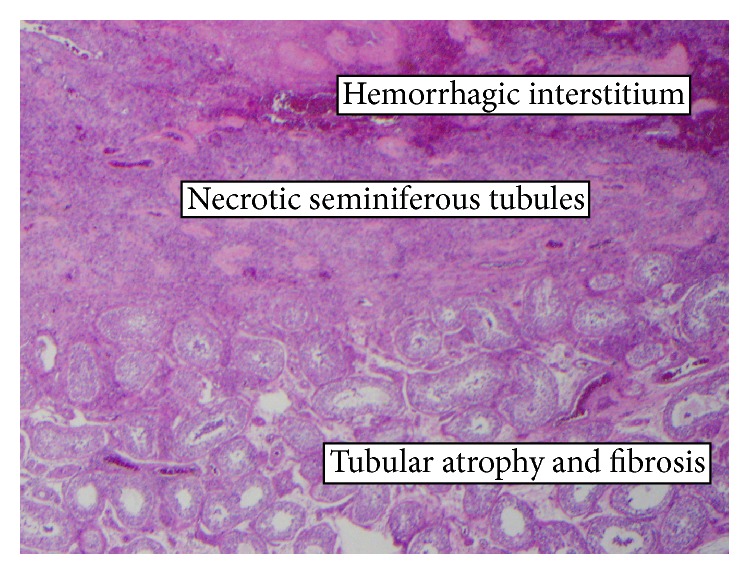
Section shows a well-defined area of infarction surrounded by an area of fibrosis and tubular atrophy. Outlines of the tubules are remaining but loss of nuclear details and hemorrhagic with hemorrhagic interstitium. There is no significant inflammation or evidence of neoplasm.

A 30-year-old male with history of motor vehicle accident leading to urinary frequency and occasional incontinence 1.5 years ago presented with 1-day history of left groin pain, which was gradually improving. On physical examination, he had a tender left testicle and epididymis with no significant swelling or systemic signs of inflammation. High frequency ultrasound including color-Doppler showed an avascular, heterogeneous, hypoechoic lesion in the superior left testis suggestive of infarction or neoplasm. Bilateral microlithiasis was also noted (Figure 1). The patient was initially managed conservatively and was also started on antibiotics for possible epididymoorchitis. Tumor markers, including Alfa-1 fetoprotein and beta HCG, were negative. His pain continued, however, and follow-up ultrasound 6 days later showed a more conspicuous lesion with interval enlargement of the hypoechoic center of the mass (Figure 2). The patient underwent left radical orchiectomy. Pathology result showed segmental infarction of the left testis (Figure 3).

## 3. Discussion

The unique anatomical location of testis, lying within an external body sac while hanging from its vascular pedicle, makes it hypermobile and prone to vascular accidents compared to other body organs. Congenital abnormalities like “bell-clapper” deformity with abnormal attachment of the testis to its muscular and facial layers increase the chance of testicular ischemic events [1, 2]. Compression of the spermatic cord as a result of torsion leads to vascular insufficiency and testicular infarction. Global testicular infarction can be easily detected on color-Doppler ultrasound by poor or absent blood flow to the testis [3], but segmental testicular infarction (STI) remains a challenging diagnosis.

Although the majority of cases with STI are idiopathic, conditions like vasculitis, sickle cell disease, polycythemia, epididymitis, intimal fibroplasia of spermatic artery, hypersensitivity angiitis, trauma, or prior testicular torsion can predispose to this condition. It can happen in any age range; however, it is a rare condition in the pediatric population [3–5]. Sudden onset testicular pain is the most common clinical presentation, which makes it difficult to differentiate from other testicular pathologies with acute onset of pain. Testicular tumor markers like Alfa-1 fetoprotein and *β*-HCG can help to differentiate it from a malignant process.

Ultrasound is usually the first step to diagnosis; however, seminomatous and nonseminomatous testicular masses can easily mimic the STI appearance on ultrasound.

On gray-scale imaging, a wedge-shaped hypoechoic lesion with the apex pointing to the rete testis can be suggestive of arterial infarction. Arterial infarctions are more often seen in the upper poles of the testes [6, 7]. A rounded pattern is more related to venous infarction, which is commonly secondary to epididymitis or germ cell tumors [3]. Abscesses are also usually round, avascular, and related to epididymitis [8]. Color-Doppler ultrasound has an important role to differentiate these entities and can prevent unnecessary orchiectomy. Absence of vascularity is highly suggestive of infarction or abscess in contrast to testicular tumors or focal orchitis, which are usually hypervascular. Some tumors, however, may be hypovascular or small, making the differentiation very difficult or impossible [6]. Newer techniques such as contrast-enhanced ultrasound with microbubble injection can potentially distinguish the tumors by showing abnormal pattern of neovascularization within the lesion. Presence of perilesional rim enhancement has also been suggested as a sign of subacute infarction indicating histologic inflammatory responses to infarction [6]. Abscesses may also have a hypervascular rim, but they are usually anechoic or very hypoechoic structures with increased through transmission and are not usually confined to a lobular distribution [6].

Magnetic resonance imaging (MRI) has also occasionally been used to aid in diagnosis. MRI can better show the lesion borders and the T2-weighted images can show low intensity signal (but can be variable) in patients with infarction. The surrounding enhanced rim can also be seen in MRI after administration of contrast [9, 10].

Despite the available imaging modalities, the diagnosis of segmental testicular infarction remains challenging. The radiologic-pathologic correlation in most case series remains suboptimal and definitive diagnosis in many patients is obtained after orchiectomy [4, 11]. When the diagnosis is made based on clinical data, most patients improve with conservative management [7, 11, 12]. In the absence of tumor markers or low clinical suspicion of malignancy, postponing surgery with longer-term follow-up should be considered to avoid unnecessary orchiectomy. Surgery may still be needed for patients who show progression of the disease or other concerning findings [4, 5, 11].

## 4. Conclusion

Acute onset of testicular pain with normal levels of tumor and inflammatory markers and presence of a wedge-shaped, avascular hypoechoic heterogenous lesion on color-Doppler ultrasound can be highly suggestive of segmental testicular infarction. The clinical and radiographic aspects of a case should be considered altogether to avoid unnecessary orchiectomy.
